# Predictors of In-Hospital Mortality in Aboriginal Children Admitted to a Tertiary Paediatric Hospital

**DOI:** 10.3390/ijerph16111893

**Published:** 2019-05-29

**Authors:** Rebecca Singer, Karen Zwi, Robert Menzies

**Affiliations:** 1 School of Public Health, Faculty of Medicine, University of New South Wales, Sydney 2033, Australia; r.menzies@unsw.edu.au; 2 School of Women’s and Children’s Health, Faculty of Medicine, University of New South Wales, Sydney 2033, Australia; Karen.zwi@health.nsw.gov.au; 3 Sydney Children’s Hospitals Network, Sydney 2031, Australia

**Keywords:** aboriginal, indigenous, children, infants, case fatality rate, mortality

## Abstract

*Background:* Aboriginal Australian children have higher rates of mortality at younger ages than non-Aboriginal Australian children. We aimed to (i) calculate the case fatality rate (CFR) for Aboriginal and non-Aboriginal children admitted to children’s hospitals in New South Wales (NSW) and (ii) identify predictors of CFR. *Methods:* We used a retrospective cross-sectional analysis of data from electronic medical records for in-patient admissions to the Sydney Children’s Hospitals Network (SCHN) over five years (2011–2015). Logistic regression analysis was used to identify predictors of mortality and excess deaths in Aboriginal children were calculated. *Results:* There were 241,823 presentations over the 5-year period. The CFR for Aboriginal children was double that of non-Aboriginal children (0.4% vs. 0.2%, *p* = 0.002), with Aboriginal children under 2 years and from remote and regional Australia at highest risk of excess mortality. Predictors of death for all children in order of significance were: Circulatory disorders (Odds Ratio (OR) 17.16, *p* < 0.001), neoplasm/blood/immune disorders (OR 2.77, *p* < 0.001), emergency admissions (OR 1.94, *p* < 0.001), aboriginality (OR 1.73, *p* = 0.005) and longer length of stay (OR 1.012; *p* < 0.001). *Conclusions:* Our data show that Aboriginal children are almost twice as likely to die than non-Aboriginal children. In particular, excess deaths in Aboriginal children are most commonly from outer regional and remote areas and children aged under 2 years with perinatal or circulatory conditions.

## 1. Introduction

There is no shortage of data showing poorer health outcomes in Aboriginal and Torres Strait Islander peoples (hereafter referred to as Aboriginal, given the low numbers of Torres Strait Islander peoples in New South Wales (NSW)) in Australia [1]. NSW is the state with the highest absolute number of Aboriginal people and children in Australia, with a population of 88,000 aged under 18 in the 2016 census [2,3]. Aboriginal Australians, in general, are younger than the general Australian population with 35% of Aboriginal peoples aged under 14 years in 2001 [4].

For those unfamiliar with the Australian context, since colonisation by the British in 1788 Australian Aboriginals have experienced massive population decline due to introduced diseases and frontier conflict, and land dispossession. Until the 1960s, policies of segregation, institutionalisation, the banning of language and cultural practices and the removal of children (known as the stolen generations), were still in existence [5,6]. Even to the present day, there is little recognition of Aboriginal Australians as the traditional owners of land and there are no formal treaties protecting rights, like those that exist in New Zealand and Canada [7]. These conditions have resulted in a legacy of intergenerational grief, with flow-on effects, such as the absence of self-determination, feelings of powerlessness, marginalisation and major disparities between Aboriginal and non-Aboriginal Australians in housing, employment, education and health [8]. Historical trauma and its intergenerational effects have resulted in poverty, increased interpersonal violence, [9] and increased rates of juvenile justice. Health effects include diseases of poverty, disproportionately high rates of suicide and addiction, and child protection involvement [10].

Additionally, racism is still a prevalent issue for Australian Aboriginals—even in 2011, almost a third of Australians continued to exhibit racist attitudes [11]. The concept of institutional racism in Australia’s health care system is a controversial one, but there is no question that there is inbuilt discrimination and disadvantage—with the Australian Medical Association stating that sub-optimal access to healthcare for Aboriginals is institutionalised inequity and is a direct contributor to poorer health outcomes [12]. Self-reported racism and chronic discrimination has a strong correlation with poorer mental and physical health outcomes, which may be mediated through increased stress and risky health behaviours, decreased use of preventative health services and poorer relationships between the patient and healthcare provider [11].

These complex historical, environmental and epigenetic factors affect the health and wellbeing of Aboriginal children [13] .They are admitted to hospital at higher rates than their non-Aboriginal peers [13,14], with more severe disease, requiring intensive care [4,15]. Aboriginal children have higher mortality rates, accounting for 10% of childhood deaths in NSW, but only accounting for 5.3% of children [16]. Aboriginal children have higher risks of dying from infective causes [13,14], respiratory causes and external causes, such as poisoning and trauma [13]. There is a paucity of data on whether these increased child mortality rates are due to increased disease incidence, later presentations, earlier discharges, a combination of these or other contributing factors. General predictors for mortality include severity at presentation, comorbid conditions—including haematological, immunological and cardiac conditions [17]—discharge against medical advice [18], malnutrition [19,20], overcrowding [19], and partial immunisation [19].

Optimisation of health and wellbeing in childhood has a positive effect on mortality, long-term morbidity, as well as educational attainment, and assists in overcoming the effects of disadvantage early in life [14]. The Australian Close the Gap initiative, commenced in 2007, aims to eliminate the child mortality gap between Aboriginal and non-Aboriginal children. National programs funded to support this include improving antenatal, maternal and child health care, parenting support, health checks, and integrated early childhood health and education services. Interventions have specifically targeted maternal smoking, alcohol consumption, antenatal attendance, low birth weight and child health surveillance, and improvements have been demonstrated in these areas [21]. Good antenatal care can reduce rates of perinatal mortality and can lower rates of preterm birth and low birth weight, even in remote settings in the Northern Territory of Australia [22]. Nationally Indigenous child mortality (aged < 5 years) reduced by 35% between 1998 and 2016, with a narrowing of the gap between Aboriginal and non-Aboriginal early childhood mortality by 33% [21,23].

NSW has the largest absolute numbers of Aboriginal children, the lowest Aboriginal child mortality rate and the smallest gap between Aboriginal and non-Aboriginal children [23,24]. There is currently no published evidence from NSW tertiary children’s hospitals regarding mortality rates and associated factors for Aboriginal children. Using a retrospective cross-sectional analysis of routinely collected data from electronic hospital medical records, we aimed to calculate the case fatality rate (CFR) for Aboriginal and non-Aboriginal children at two tertiary children’s hospitals in Sydney over a period of 5 years. We aimed to identify factors that differ between Aboriginal and non-Aboriginal children who die in tertiary children’s hospitals. This will assist in identifying, at admission, children who are at higher risk of in-hospital mortality and developing policies and tangible interventions in order to reduce this gap in mortality. This research contributes to understanding and addressing the persistence of the gap between Aboriginal and non-Aboriginal child deaths.

## 2. Materials and Methods

### 2.1. Study Setting and Design

This was a retrospective cross-sectional analysis from data extracted from the electronic medical records for in-patient admissions to Sydney Children’s Hospital Network (SCHN). This network is the largest paediatric healthcare service in Australia and provides care for the majority of children residing in NSW. It includes Sydney Children’s Hospital Randwick (SCH) and the Children’s Hospital at Westmead (CHW).

### 2.2. Participants

This study comprised all inpatient admissions to SCHN between 1 January 2011 and 31 December 2015, inclusive. Non-admitted patients presenting to the emergency and outpatients units were not included.

### 2.3. Patient and Admission Characteristics

Demographic and clinical characteristics were extracted from Citrix PowerChart Electronic Medical Records database (EMR).

The demographics extracted included the age, Aboriginality, sex, local health district and postcode of residence. The postcode was used to determine the rurality and estimate the relative socioeconomic status. The patient age was classified into: Neonate (0–28 days), infant (29 days–23 months), young child (2–5 years), child (6–12 years), adolescent (13–17 years) and adult (18–24 years). Patients aged over 25 years were excluded. Given the small number of Torres Strait Islander individuals in NSW, Aboriginality was classified as ‘Aboriginal’ (Aboriginal and/or Torres Strait Islander) and ‘not Aboriginal’. The postcode and the Australian Bureau of Statistics’ Index of Relative Socioeconomic Disadvantage (IRSD) was used to estimate socioeconomic status and disadvantage. The IRSD scores were binned into low (400–850), medium (850–1000) and high (1000–1150). The postcode was also used to determine the remoteness, classified according to the ARIA+ grading of very remote, remote, outer regional, inner regional and major cities of Australia.

Extracted clinical characteristics included urgency on admission and ICD-10 code associated with admission. The primary diagnosis was determined using the ICD-10 code. The diagnosis was grouped into broad categories by the first letter of the ICD code, for instance, codes A and B were classified as ‘Infection’. ICD-10 codes were also used to identify potentially preventable hospitalisations, classified as diagnoses for which admission could be prevented through adequate access to primary health care, as well as environmental factors such as housing, childcare and income support [25]. Deaths were identified by the mode of separation data for ‘Death—with autopsy’, and ‘Death—without autopsy’. Other modes of separation were classified as non-deaths.

### 2.4. Data Handling and Statistical Analysis

The available data were limited to characteristics identified above. Data were de-identified and stored securely under a password and available only to approved members of the research team. All demographics and clinical characteristics were analysed for each individual admission. The demographics and clinical characteristics were assessed for significant differences between Aboriginal and non-Aboriginal patients using crosstabs and a chi-square test. The case fatality rate (CFR) was calculated as the rate of deaths per 100 admissions and presented as a percentage. This was calculated for the total population deaths as well as Aboriginal and non-Aboriginal deaths. The CFR ratio was calculated by comparing the CFR of Aboriginal and non-Aboriginal admissions, and the 95% confidence interval (CI) was used to identify significant differences. Excess deaths were calculated by applying the CFR for non-Aboriginal children to the Aboriginal population.

The independence of associations for factors significantly associated with death was examined using multivariable logistic regression. The results from this regression analysis were presented using the odds ratio (OR) and the 95% CI, *p*-values of less than 0.05 were considered significant.

### 2.5. Ethics

Ethics approval was granted by the Aboriginal Health and Medical Research Council (AH&MRC) NSW (763/10), the Sydney Children’s Hospital Network (SCHN) Ethics Committee and governance committee (LNR/15/SCHN/400). The study team recognizes the diversity within the Australian Aboriginal community, as well as within the Australian community in general.

## 3. Results

### 3.1. Demographic and Clinical Characteristics of Admissions

There were 241,823 admissions over the five-year period with a median length of stay of one day (range: 1, 826) (Table 1).

Over half of admissions were in children aged less than 5 years. The age distribution was significantly different between Aboriginal and non-Aboriginal children (*p* ≤ 0.001). The Aboriginal children were younger, with an additional peak in adolescence. There were a smaller proportion of males in Aboriginal admissions as compared with non-Aboriginal admissions (54.3% vs. 57.2%, *p* ≤ 0.001). Non-Aboriginal children accounted for most admissions (96.9% vs. 3.1%). The majority of admissions were from those who lived in major cities (88.4%). Aboriginal children were more likely to come from very remote (*p* ≤ 0.001), remote (*p* ≤ 0.001), outer regional (*p* ≤ 0.001) and inner regional (*p* < 0.001) Australia, and less likely to come from major cities (*p* ≤ 0.001). Overall, more children came from medium (42.8%) and high (52.7%) socioeconomic areas, with a small number coming from low (3.8%) socioeconomic areas and overseas (0.8%). Significantly more Aboriginal children came from low (8.6% vs. 3.6%, *p* ≤ 0.001) socioeconomic areas, and came from high socioeconomic areas at half the proportion (23.8% vs. 53.6%, *p* ≤ 0.001) of non-Aboriginal children.

Just under half of the children were admitted as an emergency (45.3%). This was lower for Aboriginal children (36.7% vs. 45.6%, *p* ≤ 0.001). A small number of children died during admission (0.2%). This occurred at double the rate (0.4%, *p* = 0.002) in Aboriginal children. Around 20% of admissions were considered potentially preventable and were lower for Aboriginal children (18.1% vs. 20%, *p* ≤ 0.001). Re-admission within 48 h of previous discharge (7.2% vs. 4.8%, *p* ≤ 0.001) was higher in Aboriginal children. The top three diagnostic code groups associated with admissions were injury/poisoning (13%), respiratory diseases (11.7%), and neoplastic/blood/immune diseases (10.3%). This was different in Aboriginal children, in whom the most common diagnostic code groups were injury/poisoning (13.2%), congenital and chromosomal diseases (12.2%), and not otherwise specified (11.0%), followed by respiratory diseases (10.3%).

### 3.2. Demographic and Clinical Characteristics of Deaths

There were 543 deaths over five-years, representing a case fatality rate (CFR) of 0.22% (Table 2). Aboriginal children had a CFR almost double that of non-Aboriginal children (0.38% vs. 0.22%, *p* = 0.008). The case fatality rate was highest in children under 2 years (2.11% for neonates and 0.23% for infants) and in adolescents (0.21%). This pattern was reflected in both Aboriginal and non-Aboriginal children, however the CFR for Aboriginal children under 2 years was higher than their non-Aboriginal peers (*p* = 0.005), whilst the CFR for children aged 2–12 years remained similar. Most notably, the CFR was double in Aboriginal as compared with non-Aboriginal infants (0.48% vs. 0.23%, *p* = 0.022). The CFRs were similar for males and females overall, but point estimates were higher in Aboriginal children of both genders. CFRs were highest in those who lived in very remote Australia (0.75%) and decreased with decreasing remoteness (0.21% in major cities). This was a similar pattern for Aboriginal children; however, they had higher CFRs across all remoteness classifications. CFRs were highest in children who came from lower socioeconomic areas and decreased with increasing socioeconomic status (low = 0.31%; medium = 0.26%; high = 0.17%). This was similar in Aboriginal children; however, Aboriginal children were 1.5 times more likely to die across all socioeconomic status bands. There was a significant difference in the CFR between non-Aboriginal and Aboriginal children from medium socioeconomic areas (OR = 1.6; 95% CI = 1.03–2.50) with six excess Aboriginal deaths in this group.

The CFR for emergency admissions was higher than for non-emergency admissions (0.28% vs. 0.17%, *p* ≤ 0.001) in all children. However, for non-emergency admissions, the CFR was significantly higher in Aboriginal children compared with non-Aboriginal children (OR = 2.3, 95% CI = 1.40–3.66). The CFR was higher for potentially preventable admissions (OR = 3.5; 95% CI = 1.06*–*11.33). The diagnostic codes associated with highest CFRs were: Circulatory disorders (2.76%), perinatal disorders (1.40%), and congenital and chromosomal disorders (0.44%). This was consistent for all children. Aboriginal children had significantly higher CFRs for both circulatory disorders (OR = 3.0, 95% CI = 1.42*–*6.36) and perinatal disorders (OR = 5.7, 95% CI = 2.28*–*14.28), compared with non-Aboriginal children.

Excess deaths in Aboriginal children were predominantly due to perinatal and circulatory causes, and more excess deaths occurred in those from outer regional areas. If the CFR for non-Aboriginal children was applied to Aboriginal children, a total of 12 excess deaths occurred in Aboriginal children over the 5-year period.

### 3.3. Factors Associated with Deaths

Aboriginal children remained more likely to die than non-Aboriginal children (OR = 1.767, 95% CI = 1.21–2.57, *p* = 0.003) even after adjustment for all variables that were associated with death: The age at admission, emergency admissions and diagnostic code (Table 3).

For all children, the factors associated with increased mortality were: Aboriginality, a longer length of stay, younger age group, emergency admissions, diagnostic codes associated with neoplasm/blood/immune system and circulatory disorders.

The factors protective against mortality were: Potentially preventable hospitalisations, diagnostic codes associated with: Digestive disorders, musculoskeletal disorders and other symptoms.

The factors not significantly associated with the CFR after adjustment for the above variables were: Gender, remoteness, socioeconomic status, readmission within 48 h, diagnostic codes associated with respiratory, congenital and chromosomal, nervous system, endocrine/nutritional/metabolic, genitourinary disorders, perinatal disorders, not otherwise specified disorders, and infections.

When Aboriginality was excluded, being from a remote or regional area was associated with higher CFRs when adjusted for other contributing factors (OR = 1.842; 95% CI = 1.195–2.838; *p* = 0.006).

## 4. Discussion

Our findings show that Aboriginal children admitted to the largest tertiary children’s hospital network in NSW were 1.7 times more likely to die than non-Aboriginal children, after adjustment for age, emergency status, length of stay, and diagnostic code. The excess deaths in Aboriginal children were predominantly amongst infants and neonates, those from outer regional and remote areas, of medium socioeconomic status, and with circulatory or perinatal conditions.

We aimed to identify factors that differ between Aboriginal and non-Aboriginal children who die in tertiary children’s hospitals at admission in order to identify children at higher risk of in-hospital mortality and intervene to reduce mortality risk. Once the child has arrived in the tertiary setting, awareness of the increased risk of death in the young Aboriginal child from a non-urban setting presenting with circulatory or perinatal conditions could act as a high-risk predictive alert to healthcare staff. More frequent hospital checks, family activated systems to call for urgent help when a child deteriorates, allocation of high-risk patients to more experienced teams or a lower threshold for intensive care monitoring may assist in reducing mortality. However, whether or not these will be effective requires further research. Other interventions at the hospital level to help reduce the inequity in health status may include early involvement of Aboriginal health workers to reduce discharge against medical advice and subsequent representation, routine screening for common co-morbidities (such as anaemia and chronic infections), post-discharge linking children and their families with their local services and providing ongoing management guidance, financial assistance to purchase discharge medications, and assertive follow-up to ensure compliance.

This study also suggests that Aboriginal children are less likely to be admitted to NSW tertiary hospitals than non-Aboriginal children. The proportion of our study sample that identified as Aboriginal (3.1%) was lower than the proportion of NSW children identifying as Aboriginal (5.7%) over the time period studied. This may be explained by Aboriginal children being less likely to live in major cities in close proximity to an urban tertiary hospital and more likely to initially present to a regional or district hospital. Other studies have shown that Aboriginal children admitted to tertiary children’s hospitals are more likely to have been transferred from regional or district hospitals with higher degrees of disease severity, and increased mortality [6].

Australia has significant challenges in servicing a widely dispersed rural population [26,27,28]. Aboriginal children from outer regional and remote areas constitute 16% of our study sample, but 38% of deaths. This disproportionate CFR suggests that higher mortality may be as a result of later presentation or delayed transfers from regional or district hospitals. If this is the case, then urban tertiary children’s hospitals could play an important state-wide role in training regional and rural hospital staff in timing and appropriateness of transferring to major tertiary centres.

While later presentation with more severe disease is likely, differential treatment within the hospital is also possible. Institutional racism is well described in Australia [11,29,30,31,32,33,34,35]. Inferior care after admission has been documented as more common in Aboriginal Australians [36] and cannot be excluded as a contributing factor to the higher CFRs in Aboriginal children in our study. Increased discharge against medical advice, a marker of acceptability of the service to patients, has been shown to occur in these hospitals and may contribute to health inequity [37].

Our findings are consistent with other studies showing that perinatal and congenital conditions are the leading cause of death in infants and the second leading cause of death in all children aged 1–4 years of age [38]. Causes of death related to perinatal and circulatory causes pose a particular mortality burden to Aboriginal children. Poorer access to primary health care and specialised services is a known contributor to health differentials for those living in rural and remote Australia [27]. Infants born to Aboriginal mothers and mothers who reside in remote areas are known to have higher perinatal death rates [39]. In comparison with Aboriginal populations worldwide, New Zealand Maori [40], Canadian First Nations [41] and Native American/Native Alaskan [42] children all have higher all-cause mortality rates than their non-Aboriginal peers. These rates ranged from 1.61 times higher for Native American/Native Alaskan infants [42] to 3.6 times higher for female Canadian First Nation children [41]. However, we were unable to find appropriate comparative data on all-cause in-hospital mortality of children.

This study was a retrospective analysis of routinely collected hospitalisation data at two tertiary institutions and was, therefore, limited by data quality and availability of data from the whole of NSW. Although it is hypothesized that later presentation and greater disease severity are a major contributor to increased mortality in this population, the nature of our data meant we were unable to explore this further. Disease severity on presentation could not be reliably categorised due to the lack of granular information regarding severity within the ICD diagnostic categories. It was also not feasible to identify those children who were transferred to tertiary children’s hospitals as opposed to those presenting directly. More research is needed on the severity at presentation and specific issues related to the transfer of Aboriginal children from remote areas. A comparison between Aboriginal and non-Aboriginal children from regional and remote areas specifically would be useful to explore the relative impact of rurality and Aboriginality, but our numbers were too small to undertake this.

We were unable to identify additional factors (apart from age, rurality and certain conditions) associated with death within the Aboriginal cohort as the numbers were too small. A further limitation was the exclusion of deaths occurring in the neonatal intensive care unit at one of the two hospitals, thus under-reporting the impact of perinatal mortality in this population. For children aged 0–4 years, where comparable data exists, our study population included only 7% of NSW hospital admissions and approximately 14% of deaths in Aboriginal children in this age group that occurred in NSW during that period. Compared to all NSW hospital admissions, our sample is likely to include a greater proportion of severe or complicated cases requiring a transfer from regional hospitals and does not include deaths, which occurred prior to admission or transfer. The data used was from the two largest of three tertiary paediatric hospitals in NSW, which was the only data available under the terms of the ethics approval granted, as they form part of a single legal entity. This study is primarily aimed at identifying factors that may reduce the risk of mortality in the tertiary hospital setting, where the most complicated paediatric admissions are referred. Adding the third smaller hospital would add little additional statistical power.

## 5. Conclusions

Our data show that Aboriginal children are almost twice as likely to die than non-Aboriginal children in our tertiary children’s hospitals network in NSW. Other risk factors for Aboriginal child death include being from outer regional and remote areas and aged under 2 years with perinatal or circulatory conditions. Although there may be interventions that will impact on Aboriginal mortality within hospitals, it is likely that greater effectiveness lies within the realm of prevention of disease at the community level through public health and social policy measures, and addressing the social determinants of health (such as housing, employment, and education).

## Figures and Tables

**Table 1:** Demographics and clinical characteristics of admissions.

Clinical Characteristics	Total Admitted Children (Range)	Aboriginal Admissions (Range)	Non-Aboriginal Admissions (Range)	*p*-Value ^1^
Length of Stay (Median, Days)	1.00 (1, 826)	1.00 (1, 341)	1.00 (1, 826)	<0.001
	Total (% of Total)	Aboriginal Admissions (% of Deaths)	Non-Aboriginal Admissions (% of Total)	*p*-Value ^1^
**Age Group**				<0.001
Neonate (0–28 days)	6273 (2.5%)	237 (3.8%)	6033 (2.5%)	
Infant (29 days–23 months)	62,337 (25.3%)	2072 (27.2%)	60,213 (25.2%)	
Young Child (2–5 years)	70,080 (28.4%)	1837 (24.1%)	68,192 (28.6%)	
Child (6–12 years)	69,017 (28.0%)	2056 (27%)	66,912 (28.0%)	
Adolescent (13–17 years)	37,380 (15.2%)	1393 (18.3%)	36,039 (15.1%)	
Adult (18–25 years)	1273 (0.5%)	28 (0.4%)	1274 (0.5%)	
**Gender**				<0.001
Male	140,796 (57.1%)	4136 (54.3%)	136,616 (57.2%)	
Female	105,567 (42.9%)	3487 (45.7%)	102,047 (42.8%)	
**Aboriginality**				
Identify as Aboriginal and/or Torres Strait Islander	7625 (3.1%)			
Do not identify as Aboriginal and/or Torres Strait Islander	238,738 (96.9%)			
**Remoteness**				<0.001
Very Remote Australia	268 (0.1%)	96 (1.3%)	172 (0.1%)	
Remote Australia	700 (0.3%)	248 (3.3%)	448 (0.2%)	
Outer Regional Australia	5390 (2.2%)	935 (12.3%)	4447 (1.9%)	
Inner Regional Australia	20,324 (8.2%)	1415 (18.6%)	17,982 (7.6%)	
Major Cities of Australia	217,804 (88.4%)	4919 (64.6%)	213,746 (90.3%)	
Other/Overseas	1877 (0.8%)	12 (0.2%)	1943 (0.8%)	
**IRSD Category**				<0.001
Low (<850)	9274 (3.8%)	658 (8.6%)	8605 (3.6%)	
Med (850–1000)	105,348 (42.8%)	5112 (67.1%)	100,054 (41.9%)	
High (>1000)	129,682 (52.7%)	1814 (23.8%)	127,896 (53.6%)	
N/A (e.g., overseas)	1879 (0.8%)	39 (0.5%)	2108 (0.9%)	
**Urgency at Admission**				<0.001
Emergency	111,709 (45.3%)	2800 (36.7%)	108,882 (45.6%)	
Non-Emergency	134,621 (54.7%)	4823 (63.3%)	129,781 (54.4%)	
**Death During Admission**				0.002
Yes	543 (0.2%)	29 (0.38%)	513 (0.2%)	
No	246,820 (99.8%)	7594 (99.6%)	238,150 (99.8%)	
**Potentially Preventable Hospitalisation**				<0.001
No	197,284 (80.1%)	6247 (81.9%)	191,015 (80%)	
Yes	49,028 (19.9%)	1376 (18.1%)	47,648 (20%)	
**Readmission within 48 h**				<0.001
No	234,333 (95.1%)	7073 (92.8%)	227,234 (95.2%)	
Yes	11,979 (4.9%)	550 (7.2%)	11,429 (4.8%)	
**Main Diagnostic Code**				
Infection	10,681 (4.3%)	248 (3.3%)	10,433 (4.4%)	<0.001
Neoplasm/Blood/Immune	25,313 (10.3%)	548 (7.2%)	24,763 (10.4%)	<0.001
Endocrine/Nutritional/Metabolic	8208 (3.3%)	377 (4.9%)	7831 (3.3%)	<0.001
Mental & Behavioural	2603 (1.1%)	92 (1.2%)	2511 (1.1%)	0.193
Nervous System	14,163 (5.8%)	554 (7.3%)	13,608 (5.7%)	<0.001
Eye, ear & adnexa	7528 (3.1%)	343 (4.5%)	7183 (3.0%)	<0.001
Circulatory	2679 (1.1%)	90 (1.2%)	2589 (1.1%)	0.427
Respiratory	28,715 (11.7%)	782 (10.3%)	27,930 (11.7%)	<0.001
Digestive	19,856 (8.1%)	487 (6.4%)	19,366 (8.1%)	<0.001
Skin	7064 (2.9%)	236 (3.1%)	6828 (2.9%)	0.226
Musculoskeletal	9561 (3.9%)	267 (3.5%)	9293 (3.9%)	0.082
Genitourinary	9724 (3.9%)	288 (3.8%)	9435 (4.0%)	0.439
Pregnancy & Childbirth	5 (<1%)	1 (<0.1%)	3 (<0.1%)	0.011
Perinatal	2503 (1%)	71 (0.9%)	2432 (1.0%)	0.453
Congenital & chromosomal	22,726 (9.2%)	927 (12.2%)	21,794 (9.1%)	<0.001
Other symptoms	21,234 (8.6%)	474 (6.2%)	20,757 (8.7%)	<0.001
Injury/poisoning	31,918 (13%)	1003 (13.2%)	30,914 (13.0%)	0.601
Not Otherwise Specified	21,831 (8.9%)	835 (11.0%)	20,993 (8.8%)	<0.001

^1^ Aboriginal compared to non-Aboriginal Admissions.

**Table 2:** Demographic and clinical characteristics of deaths.

Clinical Characteristics	In-Hospital Deaths (Range)	Aboriginal Deaths (Range)	Non-Aboriginal Deaths (Range)	*p*-Value ^#^	CFR ^ Ratio (95% CI)
Median Length of Stay (Days)	5.00 (1, 404)	3.00 (1, 241)	5.00 (1, 404)	0.568	
	Deaths (CFR)	Aboriginal Deaths (CFR)	Non-Aboriginal Deaths (CFR)	*p*-Value ^#^	
**Age Group**				0.576	
Neonate (0–28 days)	135 (2.11%)	8 (3.38%)	127 (2.11%)		1.6 (0.79–3.24)
Infant (1 to 23 months)	148 (0.23%)	10 (0.48%)	138 (0.23%)		2.1 (1.11*–*3.99) *
Young Child (2–5 years)	99 (0.14%)	3 (0.16%)	96 (0.14%)		1.2 (0.37*–*3.66)
Child (6–12 years)	78 (0.11%)	2 (0.10%)	76 (0.11%)		0.9 (0.21*–*3.48)
Adolescent (13–17 years)	80 (0.21%)	6 (0.43%)	74 (0.21%)		2.1 (0.91*–*4.81)
Adult (18 years or more)	3 (0.24%)	0 (0.0%)	3 (0.24%)		
**Gender**				0.846	
Male	309 (0.23%)	16 (0.39%)	293 (0.21%)		1.8 (1.09*–*2.98) *
Female	234 (0.22%)	13 (0.37%)	221 (0.22%)		1.7 (0.98*–*3.01)
**Aboriginality**					1.7 (1.21–2.57) *
Aboriginal	29 (0.38%)				
Non-Aboriginal	514 (0.22%)				
**Remoteness**				<0.001	
Very Remote Australia	2 (0.75%)	1 (1.04%)	1 (0.58%)		1.8 (0.11*–*28.32)
Remote Australia	4 (0.57%)	3 (1.21%)	1 (0.22%)		5.4 (0.57*–*52.82)
Outer Regional Australia	17 (0.32%)	8 (0.86%)	9 (0.20%)		4.2 (1.63*–*10.93) *
Inner Regional Australia	50 (0.25%)	4 (0.28%)	46 (0.26%)		1.1 (0.40*–*3.06)
Major Cities of Australia	459 (0.21%)	13 (0.26%)	446 (0.21%)		1.3 (0.73*–*2.20)
Other/Overseas	11 (0.59%)	0 (0.0%)	11 (0.57%)		
**IRSD Category**				0.027	
Low (<850)	29 (0.31%)	3 (0.46%)	26 (0.30%)		1.5 (0.46*–*4.97)
Med (850–1000)	277 (0.26%)	21 (0.41%)	256 (0.26%)		1.6 (1.03*–*2.50) *
High (>1000)	225 (0.17%)	5 (0.28%)	220 (0.17%)		1.6 (0.66*–*3.88)
N/A (e.g., overseas)	12 (0.64%)	0 (0.0%)	12 (0.57%)		
**Urgency at Admission**				0.030	
Emergency	311 (0.28%)	11 (0.39%)	300 (0.28%)		1.4 (0.78*–*2.60)
Non-Emergency	232 (0.17%)	18 (0.37%)	214 (0.16%)		2.3 (1.40*–*3.66) *
**Potentially Preventable Hospitalisation**				0.409	
No	510 (0.26%)	26 (0.42%)	484 (0.25%)		1.6 (1.11*–*2.43) *
Yes	33 (0.07%)	3 (0.22%)	30 (0.06%)		3.5 (1.06*–*11.33) *
**Readmission within 48 h**				1.000	
No	524 (0.22%)	28 (0.40%)	496 (0.22%)		1.8 (1.24*–*2.65) *
Yes	19 (0.16%)	1 (0.18%)	18 (0.16%)		1.2 (0.15*–*8.63)
**Main Diagnostic Code °**					
Infection	27 (0.25%)	1 (0.40%)	26 (0.25%)	1.000	1.6 (0.22*–*11.88)
Neoplasm/Blood/Immune	83 (0.33%)	2 (0.36%)	81 (0.33%)	0.289	1.1 (0.27*–*4.53)
Endocrine/Nutritional/Metabolic	11 (0.13%)	0 (0.0%)	11 (0.14%)	1.000	
Mental & Behavioural	0 (0%)	0 (0.0%)	0 (0%)		
Nervous system	42 (0.30%)	2 (0.36%)	40 (0.29%)	1.000	1.2 (0.30*–*5.07)
Eye, ear & adnexa	0 (0%)	0 (0.0%)	0 (0%)		
Circulatory	74 (2.76%)	7 (7.78%)	67 (2.59%)	0.003	3.0 (1.42*–*6.36) *
Respiratory	37 (0.13%)	1 (0.13%)	36 (0.13%)	0.712	1.0 (0.13*–*7.23)
Digestive	11 (0.06%)	0 (0.0%)	11 (0.06%)	1.000	
Skin	0 (0%)	0 (0.0%)	0 (0%)		
Musculoskeletal	3 (0.03%)	0 (0.0%)	3 (0.03%)	1.000	
Genitourinary	2 (0.02%)	1 (0.35%)	1 (0.01%)	0.104	32.8 (2.05*–*522.46) *
Pregnancy & Childbirth	0 (0%)	0 (0.0%)	0 (0%)		
Perinatal	35 (1.40%)	5 (7.04%)	30 (1.23%)	<0.001	5.7 (2.28*–*14.28) *
Congenital & chromosomal	99 (0.44%)	4 (0.43%)	95 (0.44%)	0.525	1.0 (0.36*–*2.69)
Other symptoms	18 (0.08%)	1 (0.21%)	17 (0.08%)	1.000	2.6 (0.34*–*19.31)
Injury/poisoning	63 (0.20%)	4 (0.40%)	59 (0.19%)	0.763	2.1 (0.76*–*5.74)
Not Otherwise Specified	38 (0.17%)	1 (0.12%)	37 (0.18%)	0.712	0.7 (0.09*–*4.95)

^#^ Aboriginal compared with Non-Aboriginal; * and bold denotes significant finding; ^ case fatality rate; ° no reference category available as compared against all other admissions (e.g., infection diagnosis compared with non-infection diagnoses).

**Table 3:** Factors associated with death (multivariate logics regression) in all children.

Characteristic		OR	95% CI	*p*-Value
Length of Stay	Days	1.014	1.012*–*1.016	<0.001
Age Group (c.f. neonates)				<0.001
	Neonates (0*–*28 days)	ref		
	Infant (1*–*23 months)	0.124	0.097*–*0.158	<0.001
	Young Child (2*–*5 years)	0.071	0.054*–*0.094	<0.001
	Child (6*–*12 years)	0.046	0.042*–*0.075	<0.001
	Adolescent (13*–*17 years)	0.105	0.078*–*0.140	<0.001
	Adult (>18y)	0.140	0.044*–*0.444	0.001
Aboriginality	Aboriginal	1.727	1.178*–*2.532	0.005
	Non-Aboriginal	ref		
Emergency Admission	Emergency Admission	1.940	1.619*–*2.325	<0.001
	Non-Emergency Admission	ref		
Main Diagnostic Code	Neoplasm/Blood/Immune	2.765	2.140*–*3.571	<0.001
	Circulatory	17.158	13.190*–*22.319	<0.001
	Digestive	0.328	0.174*–*0.619	0.001
	Musculoskeletal	0.257	0.082*–*0.805	0.020
	Other Symptoms	0.398	0.247*–*0.642	<0.001

## References

[1] Heuvel A.V. (2015). The Health and Welfare of Australia’s Aboriginal and Torres Strait Islander Peoples, 2015.

[2] Al-Yaman F., Bryant M., Sargeant H. (2001). Australia’s Children: Their Health and Wellbeing 2002: The First Report on Children’s Health by the Australian Institute of Health and Welfare.

[3] Australian Bureau of Statistics (2017). 2016 Census QuickStats (Australia).

[4] Eades S.J., Stanley F.J. (2013). Improving the health of First Nations children in Australia. Med. J. Australia.

[5] Haebich A. (2000). Broken Circles: Fragmenting Indigenous Families, 1800–2000.

[6] Whiteside M., Tsey K., Earles W. (2011). Locating empowerment in the context of Indigenous Australia. Aust. Soc. Work.

[7] Tilbury C. (2009). The over-representation of indigenous children in the Australian child welfare system. Int. J. Soc. Welf..

[8] Trewin D., Madden R. (2005). The Health and Welfare of Australia’s Aboriginal and Torres Strait Islander Peoples.

[9] Paradies Y. (2016). Colonisation, racism and indigenous health. J. Pop. Res..

[10] Cunneen C., Libesman T. (2000). Postcolonial trauma: The contemporary removal of Indigenous children and young people from their families in Australia. Aust. J. Soc. Issues.

[11] Awofeso N. (2011). Racism: A major impediment to optimal Indigenous health and health care in Australia. Aust. Indigenous Health Bull..

[12] Australian Medical Association (2007). Institutionalised Inequity, Not Just A Matter of Money.

[13] Carville K.S., Lehmann D., Hall G., Moore H., Richmond P., de Klerk N., Burgner D. (2007). Infection is the major component of the disease burden in aboriginal and non-aboriginal Australian children: A population-based study. Pediat. Inf. Dis. J..

[14] Freemantle J., McAullay D. (2009). Indigenous Children’s Health Report: Health Assessment in Action.

[15] Ostrowski J.A., MacLaren G., Alexander J., Stewart P., Gune S., Francis J.R., Ganu S., Festa M., Erickson S.J., Straney L. (2017). The burden of invasive infections in critically ill Indigenous children in Australia. Med. J. Australia.

[16] NSW Child Death Review Team (2016). Child Death Review Report 2015.

[17] Vendetti N., Zaoutis T., Coffin S.E., Sammons J.S. (2015). Risk factors for in-hospital mortality among a cohort of children with Clostridium difficile infection. Infect. Control Hosp. Epidemiol..

[18] Southern W.N., Nahvi S., Arnsten J.H. (2012). Increased risk of mortality and readmission among patients discharged against medical advice. Amer. J. Med..

[19] Savitha M.R., Nandeeshwara S.B., Kumar M.J.P., Raju C.K. (2007). Modifiable risk factors for acute lower respiratory tract infections. Indian J. Pediat..

[20] Tette E.M.A., Nyarko M.Y., Nartey E.T., Neizer M.L., Egbefome A., Akosa F., Biritwum R.B. (2016). Under-five mortality pattern and associated risk factors: A case-control study at the Princess Marie Louise Children’s Hospital in Accra, Ghana. BMC Pediatr..

[21] Australian Health Ministers’ Advisory Council (2017). Aboriginal and Torres Strait Islander Health Performance Framework 2017 Report.

[22] Kildea S., Kruske S., Barclay L., Tracy S. (2010). ‘Closing the Gap’: How maternity services can contribute to reducing poor maternal infant health outcomes for Aboriginal and Torres Strait Islander women. Rural Remote Health.

[23] Department of the Prime Minister and Cabinet (2018). Closing The Gap: Prime MInister’s Report 2018.

[24] Gardner S., Woolfenden S., Callaghan L., Allende T., Winters J., Wong G., Caplice S., Zwi K. (2016). Picture of the health status of Aboriginal children living in an urban setting of Sydney. Aust. Health Rev..

[25] Anderson P., Craig E., Jackson G., Jackson C. (2012). Developing a tool to monitor potentially avoidable and ambulatory care sensitive hospitalisations in New Zealand children. N. Z. Med. J..

[26] Wilson N., Couper I., De Vries E., Reid S., Fish T., Marais B. (2009). Inequitable distribution of healthcare professionals to rural and remote areas. Rural Remote Health.

[27] McGrail M.R., Humphreys J.S. (2015). Spatial access disparities to primary health care in rural and remote Australia. Geospatial Health.

[28] Armstrong B.K., Gillespie J.A., Leeder S.R., Rubin G.L., Russell L.M. (2007). Challenges in health and health care for Australia. Med. J. Australia.

[29] Davidson P.M., MacIsaac A., Cameron J., Jeremy R., Mahar L., Anderson I. (2012). Problems, solutions and actions: Addressing barriers in acute hospital care for indigenous Australians and New Zealanders. Heart, Lung Circ..

[30] Durey A., Thompson S.C. (2012). Reducing the health disparities of Indigenous Australians: Time to change focus. BMC Health Serv. Res..

[31] Shepherd C.C.J., Li J., Cooper M.N., Hopkins K.D., Farrant B.M. (2017). The impact of racial discrimination on the health of Australian Indigenous children aged 5–10 years: Analysis of national longitudinal data. Int. J. Equity Health.

[32] Durey A., Thompson S.C., Wood M. (2012). Time to bring down the twin towers in poor Aboriginal hospital care: Addressing institutional racism and misunderstandings in communication. Intern. Med. J..

[33] Henry B.R., Houston S., Mooney G.H. (2004). Institutional racism in Australian healthcare: A plea for decency. Med. J. Australia.

[34] Larson A., Gillies M., Howard P.J., Coffin J. (2007). It’s enough to make you sick: The impact of racism on the health of Aboriginal Australians. Aust. N. Z. Publ. Health.

[35] Paradies Y., Harris R., Anderson I. (2008). The Impact of Racism on Indigenous Health in Australia and Aotearoa: Towards a Research Agenda.

[36] Bureau of Health Information (2016). Patient Perspectives—Hospital Care for Aboriginal People.

[37] Sealy L., Zwi K., McDonald G., Saavedra A., Crawford L., Gunasekera H. (2019). Predictors of Discharge Against Medical Advice in a Tertiary Paediatric Hospital. Int. J. Environ. Res. Public Health.

[38] Australian Institute of Health and Welfare (2018). Deaths in Australia.

[39] Centre for Epidemiology and Evidence (2014). The Health of Children and Young People in NSW: Report of the Chief Health Officer 2014.

[40] Fraser J., Sidebotham P., Frederick J., Covington T., Mitchell E.A. (2014). Learning from child death review in the USA, England, Australia, and New Zealand. Lancet.

[41] Peters P.A., Oliver L.N., Kohen D.E. (2013). Mortality among children and youth in high-percentage First Nations identity areas, 2000–2002 and 2005–2007. Rural Remote Health.

[42] Wong C.A., Gachupin F.C., Holman R.C., MacDorman M.F., Cheek J.E., Holve S., Singleton R.J. (2014). American Indian and Alaska Native infant and pediatric mortality, United States, 1999–2009. Amer. J. Public Health.

